# Data-Driven Characterization of Metabolome Reprogramming during Early Development of Sorghum Seedlings

**DOI:** 10.3390/metabo14020112

**Published:** 2024-02-07

**Authors:** Ian A. Dubery, Lerato P. Nephali, Fidele Tugizimana, Paul A. Steenkamp

**Affiliations:** Research Centre for Plant Metabolomics, Department of Biochemistry, University of Johannesburg, P.O. Box 524, Auckland Park 2006, South Africa; 201495571@student.uj.ac.za (L.P.N.); ftugizimana@uj.ac.za (F.T.); psteenkamp@uj.ac.za (P.A.S.)

**Keywords:** developmental stage, metabolome, multivariate data analysis, specialized metabolite, *Sorghum bicolor*

## Abstract

Specialized metabolites are produced via discrete metabolic pathways. These small molecules play significant roles in plant growth and development, as well as defense against environmental stresses. These include damping off or seedling blight at a post-emergence stage. Targeted metabolomics was followed to gain insights into metabolome changes characteristic of different developmental stages of sorghum seedlings. Metabolites were extracted from leaves at seven time points post-germination and analyzed using ultra-high performance liquid chromatography coupled to mass spectrometry. Multivariate statistical analysis combined with chemometric tools, such as principal component analysis, hierarchical clustering analysis, and orthogonal partial least squares–discriminant analysis, were applied for data exploration and to reduce data dimensionality as well as for the selection of potential discriminant biomarkers. Changes in metabolome patterns of the seedlings were analyzed in the early, middle, and late stages of growth (7, 14, and 29 days post-germination). The metabolite classes were amino acids, organic acids, lipids, cyanogenic glycosides, hormones, hydroxycinnamic acid derivatives, and flavonoids, with the latter representing the largest class of metabolites. In general, the metabolite content showed an increase with the progression of the plant growth stages. Most of the differential metabolites were derived from tryptophan and phenylalanine, which contribute to innate immune defenses as well as growth. Quantitative analysis identified a correlation of apigenin flavone derivatives with growth stage. Data-driven investigations of these metabolomes provided new insights into the developmental dynamics that occur in seedlings to limit post-germination mortality.

## 1. Introduction

Sorghum (*Sorghum bicolor* (L.) Moench) is a food and staple crop that is indigenous to the African continent, of particular use as a grain crop in arid areas. Sorghum is cultivated for either the production of bioenergy, animal feed, and/or human consumption in over 30 countries; therefore, the production of this crop plays a significant role in the global economy, as well as in alleviating food insecurity and unemployment [1]. Sorghum seedlings are very delicate during the emergence period and extremely vulnerable to soil-borne pathogens under suboptimal growing conditions. Many fungal pathogens that are common soil inhabitants (e.g., *Rhizoctonia*, *Fusarium*, *Sclerotinia*, *Verticillium*, and *Pythium* spp.) can cause pre- and post-emergence damping-off or seedling blight. Affected seedlings may show yellowing, wilting, and death of leaves, with the roots of diseased plants discolored and rotten (https://infonet-biovision.org/plant_pests/damping-diseases, accessed on 15 December 2023). Some soil-borne diseases have been a serious problem for many decades and are responsible for restrictions on agricultural yield. Thus far, no biological control strategies have been developed [2].

The ability of a plant to overcome attempted pathogen attacks determines the level of resistance thereof. Small-molecule metabolites are closely linked to the phenotypic characteristics of a plant or cultivar. The phenotypically observable changes in the developmental trajectory of sorghum seedlings reflect the underlying metabolomic reconfigurations. Generally, the type and concentration(s) of specialized metabolites are determined by the species, genotype, physiology, developmental stage, and environmental factors during growth [3,4]. Sorghum contains various specialized phytochemicals that contribute to the nutritional properties and the development of the crop. The most predominant metabolites in sorghum plants are phenolic compounds, which include flavonoids, tannins, anthocyanins, and cinnamic acids [5]. Flavonoids and phenolic acids support crop growth in extreme environmental conditions and provide the plant with adaptive coping mechanisms to deal with both abiotic and biotic stresses. These phenolic compounds have associated antioxidant properties, known to reduce oxidative stress [6], as well as antimicrobial properties [7,8,9].

To descriptively understand developmental changes of sorghum, ‘omics’ sciences, such as metabolomics, have evolved to be indispensable in interrogating cellular biochemistry. As such, it contributes to a comprehensive characterization of the metabolome and cellular dynamics of the biological system under consideration [10,11]. Such studies contribute towards improving breeding strategies (correlating agronomical traits to a metabolic phenotype), creating stress-resilient crops, and increasing crop quality and yield [9,11]. Thus, reported herein is a liquid chromatography–mass spectrometry (LC-MS)-based metabolomics study to elucidate metabolic changes of sorghum seedlings over the early growth period following germination. Chemometrics methods, such as principal component analysis (PCA) and orthogonal partial least squares–discriminant analysis (OPLS-DA), were applied to mine and interpret the generated metabolomics datasets, elucidating differential metabolic profiles at different stages, i.e., time points post-germination. Such insights would point to the dynamics of seedling metabolism, also revealing possible biochemical events that are involved in protection and adaptation at this early growth stage of the plants.

## 2. Materials and Methods

### 2.1. Sorghum Seedling Cultivation

Seeds from the *Sorghum bicolor* cv. NS 5511, a red/bitter seed variety [9], were obtained from a commercial seed supplier, (Agricol, Pretoria, South Africa), and cultivated in germination mix soil (Culterra, Muldersdrift, South Africa). The seeds were sown in trays (23 × 36 × 6 cm) under controlled greenhouse conditions: a light/dark cycle of 12 h/12 h, an average light intensity of 85 µmol/m^2^/s, and the temperature regulated to between 22 and 24 °C. The seedlings were harvested at 7, 11, 14, 18, 22, 25, and 29 days post-germination (d.p.g.). For data analysis, days 7, 14, and 29 were designated as corresponding to ‘early’, ‘middle’, and ‘late’ developmental stages, respectively. The experimental design included three independent biological replicates of each time point. The harvested leaves were weighed, snap-frozen to quench metabolic activity, and stored at −80 °C until extraction (Supplementary Figure S1A,B).

### 2.2. Metabolite Extraction and Pre-Analytical Sample Preparation

Metabolites were extracted as previously described [9]. Briefly, frozen leaf tissue was mixed with a cold extraction solvent (80% aqueous methanol) in a ratio of 1:15 (*m*/*v*). The mixture was homogenized using an Ultra-Turrax homogenizer (CAT Scientific, Berlin, Germany), followed by sonication for 15 s with a probe sonicator (Bandelin Sonopuls, Berlin, Germany) set at 55% power. Homogenates were centrifuged at 5000× *g* and 4 °C for 25 min. The supernatants of each sample were then concentrated by evaporation under vacuum to 1 mL using a rotary evaporator set at 55 °C. The 1 mL extracts from each sample were further evaporated to complete dryness with a speed vacuum concentrator (Eppendorf, Merck, Johannesburg, South Africa) set at 45 °C. The final step of sample preparation consisted of resuspending the dried extracts in 50% LC-grade methanol (Romil, Cambridge, UK) in a 1:10 *m*/*v* ratio. This was followed by filtering samples through 0.22 µm nylon syringe filters into glass chromatography vials fitted with 500 µL inserts. The filtered extracts were capped and kept at −20 °C until analysis. 

### 2.3. Ultra-High Performance Liquid Chromatography (UHPLC) Coupled to High-Definition Mass Spectrometry (MS) and Data Processing

Analyses were performed on a Waters Acquity UHPLC connected in tandem to a SYNAPT G1 Q-TOF mass spectrometer via an electrospray ionization (ESI) interface and operated with MassLynxTM software (ver. 4.1, Waters Corporation, Manchester, UK). Sample extracts were chromatographically separated on a reverse-phase C18 column (150 mm × 2.1 mm × 1.8 µm—HSS T3, Waters Corporation, Milford, MA, USA) at 60 °C. The mobile phase consisted of 0.1% formic acid in MilliQ water (solvent A) and 0.1% formic acid in acetonitrile (Romil, Cambridge, UK) (solvent B) with a flow rate of 0.4 mL/min. Gradient elution was used, and the initial conditions were 2% B and maintained for 1 min. The gradient was ramped up to 95% B at 15 min and maintained for 2 min, and then changed to the initial conditions at 18 min, followed by a 2 min equilibration time of the column. The total chromatographic run time was 20 min, and the injection volume was 2 µL. Each sample, originating from three independent biological replicates, was analyzed in triplicate (*n* = 9) in both positive and negative ESI modes. Sample acquisition was randomized, and the quality control (QC) sample used to monitor the performance and stability of the UHPLC-MS system was repeatedly injected to evaluate any analytical variability. The conditions were set as follows: capillary voltage of 2.5 kV, sampling cone at 30 V, extraction cone at 4 V, cone gas flow of 50 L/h, desolvation gas flow of 550 L/h, source temperature at 120 °C, desolvation temperature at 450 °C, scan time of 0.1 s, and mass range of 100–1000 Da. Leucine enkephalin (50 pg/mL, [M+H]^+^ = 556.2771 Da and [M–H]^−^ = 554.2615 Da) was used as a reference calibrant at a flow rate of 0.1 mL/min. It was sampled every 15 s and produced an average intensity of 350 counts/scan in centroid mode. The mass accuracy window was 0.5 Da, with a typical mass accuracy ranging from 1 to 3 mDa.

In addition, a data-independent acquisition (DIA) method, namely MS^E^, was applied; the MS analyses were set to carry out non-fragmented as well as five fragmenting experiments simultaneously by applying alternating collision energy of 0 eV (unfragmented) and from 10 to 50 eV (fragmented). This was performed to generate molecular fragmentation data for downstream structural elucidation required for compound annotation or identification.

Pre-processing of raw MS data was performed using the MarkerLynx^TM^ application manager for MassLynx^TM^ XS software version 4.1 (Waters Corporation, Manchester, UK), for detection and alignment of peaks, as well as cleaning of data matrices for reduced noise and redundancy. 

### 2.4. Metabolite Annotation

Annotation of spectral features was based upon physicochemical properties and/or spectral similarity with public/commercial spectral libraries and according to level 2 of the Metabolomics Standards Initiative [12]. Based on accurate mass determinations from the UHPLC-MS analysis, a potential empirical formula was calculated for each peak of interest using the *m*/*z* values and searched against the databases such as PubChem (https://pubchem.ncbi.nlm.nih.gov/, accessed on 30 June 2023), the Dictionary of Natural Products (http://dnp.chemnetbase.com/faces/chemical/ChemicalSearch.xhtml, accessed on 31 May 2023), MS-DIAL (Mass Spectrometry-Data Independent Analysis software, http://prime.psc.riken.jp, accessed on 16 July 2023, version 4.9.221218) and ChemSpider (http://www.chemspider.com/, accessed on 16 July 2023), also taking possible adduct formation into account. The chemical structures were confirmed by inspecting the MSE information derived from the MS analyses at the five different fragmentation conditions. 

In addition, the generated data matrices were also processed using the Taverna workbench (www.taverna.org.uk, accessed on 5 January 2019) for PUTMEDID_LCMS identification of metabolite workflows by correlation analysis, annotation of metabolic features, and putative identification of metabolites, as previously described [10,13]. For the Taverna workbench analysis, data matrices were formatted from MarkerLynx-based data processing. The Taverna Metabolite ID procedure consists of three key workflows: (i) Pearson-based correlation analysis (List CorrData), (ii) metabolic feature annotation (annotate Massmatch), permitting the grouping of ion peaks with comparable properties like Rt, and annotating features with the type of *m*/*z* ion (molecular ion, isotope, possible adducts, etc.) assumed to be derived from the same metabolite. The elemental composition/molecular formula (MF) of each *m*/*z* ion was then computed, and (iii) metabolite annotation (matchMF-MF) of the computed MF was automatically compared and matched to the MF from a pre-defined reference list of sorghum metabolites [9,10,14].

### 2.5. Visualization and Comparison of Annotated Metabolite Trends 

A triangle/ternary plot, constructed with Microsoft Excel, was used for the overall comparison of the annotated compounds in the analyzed samples [15]. Triangle plots are graphical representations of variables that sum to a constant (100%), represented within a two-dimensional triangle. The original data were normalized to 100% and transformed into X and Y coordinates, which were then plotted on a scatter plot with coordinates for a triangle. Heatmaps support the visualization of multidimensional datasets and identify metabolic patterns under similar experimental conditions. In addition, heat maps can be used to locate hidden groups among identified metabolites and associations between experimental groups and metabolic changes [16]. Following annotation of the discriminant features, heat maps were constructed for the corresponding metabolites using the MetaboAnalyst bioinformatics tool suite (version 4.0; http://www.metaboanalyst.ca/, accessed on 3 December 2022) [17]. Average peak intensities (*n* = 9) were used to construct heat maps illustrating differences in the relative concentrations of the selected analytes from the different groups. 

### 2.6. Data Mining, Multivariate Data Analysis, and Statistical Modeling

For data mining and multivariate data statistical analysis (MVDA), annotated metabolites (43) were further analyzed using SIMCA 15 (Soft Independent Modelling of Class Analogy, including the ‘omics’ skin) (Sartorius, Stedim Data Analytics AB, Umeå, Sweden) and MetaboAnalyst 4.0 (http://www.metaboanalyst.ca/, accessed on 23 March 2022). Such analyses included data exploration and clustering. Before MVDA and computation of chemometric models, log transformation and Pareto scaling were performed on the data for variable normalization. As unsupervised methods, principal component analysis (PCA) was applied to reduce the dimensionality of the data and to obtain an overview of the metabolic data, general clustering, and trends. Hierarchical cluster analysis (HiCA) was used to analyze the natural structure and patterns within the datasets. The information derived from these unsupervised methods was used to obtain more insights by applying a supervised method, orthogonal partial least squares–discriminant analysis (OPLS-DA), as a binary classification method within a reduced dimensional space. OPLS-DA also identifies discriminant molecules specific to the different sample group classifications. Here, the comparisons included the 7 vs. 14 d, 7 vs. 29 d, and 14 vs. 29 d groups. OPLS-DA models were validated using various multivariate statistical tools and included explained variation (R^2^) and predictive ability (Q^2^) metrics, the analysis of variance testing of cross-validated predictive residuals (CV-ANOVA, *p*-value ˂ 0.05 as a cut-off), and response permutation tests (with *n* = 100) [18], as described in the legends to the figures. These MVDA models were constructed for comparison of the seedling sample datasets for all the time points post-germination. OPLS-DA S (loadings/scatter) plots were used to identify *m*/*z* features or variables with both high correlation and covariation, [p(corr) ≥ 0.5, ≤−0.5 and (p1) ≥ 0.1, ≤−0.1]. From the OPLS-DA analyses, variable importance in projection (VIP) plots were used to identify the most significantly altered metabolites extracted from the OPLS-DA models to explore their potential biological significance. VIP values > 1 were used as the cut-off for statistical significance and to avoid possible bias in feature selection.

### 2.7. Metabolomics Pathway Analysis and Network Correlation Analyses

The identified significant metabolites (with their respective Kyoto Encyclopedia of Genes and Genomes (KEGG; https://www.genome.jp/kegg/, accessed on 23 May 2022) identifiers) were uploaded into the MetPA tool for identification, analysis, and visualization of the affected metabolic pathways (MetaboAnalyst 4.0 (http://www.metaboanalyst.ca/, accessed on 10 October 2022). MetPA performs pathway topological analysis, and the possible biological roles can be inferred/evaluated through enrichment analysis [14,18]. The pathway analysis algorithms specified for over-representation analysis were the hypergeometric test, and for pathway topology analysis, it was the relative betweenness centrality [10]. The global significance of a pathway enrichment is estimated by ranking the *p*-value from real data among the *p*-values from permutation data to adjust for type I error [18]. MetPA-computed metabolic pathway analysis generates a visual representation of information showing all matched pathways according to the log *p*-values and impact scores, as shown in Table S2. As a complementary approach, the interconnectedness of the active pathways was modeled using KEGG MAPPER (https://www.genome.jp/kegg/mapper.html, accessed on 24 May 2022) by uploading KEGG identifiers of the annotated metabolites via a searcher pathway option, where compounds are searched against KEGG pathway maps. Statistical analyses are also used to describe these pathways by the *p*-values and false discovery rate (FDR) of the individual metabolites [19]. The *p*-value was set at <0.1 and the FDR cut-off was <0.5.

Network correlation analyses were developed to examine direct biochemical associations. Here, assigned KEGG identifiers of each annotated metabolite were uploaded on the KEGG mapping tool (https://www.genome.jp/kegg/tool/map_pathway1.html, accessed on 24 May 2022), using the organism-specific search mode for *Arabidopsis thaliana*. The network was visualized using the Cytoscape version 2.8.2 tool (https://cytoscape.org/, accessed on 28 May 2022), and network characteristic mapping reflected chemometric modeling information via network edge (or link) and node (or vertex) features [20]. The centrality parameter is a quantitative measure of the position of a node relative to the other nodes, commonly applied in the estimation of a node’s relative significance in network organization. Considering that metabolic networks are directed graphs, the significance role played by a compound is determined using ‘relative betweenness centrality’ and ‘out degree centrality’ in MetPA. The pathway impact is measured as the collection of the significance measures of the corresponding metabolites normalized by the sum of the significance measures of the total metabolites in each pathway.

### 2.8. Multiple Reaction Monitoring (MRM) UHPLC-MS/MS Method for the Quantification of Targeted Defense-Related Flavonoids

Pure, authentic standards were obtained from Chengdu Biopurify Phytochemicals (Chengdu, Sichuan, China), while D-fluorophenylalanine was obtained from Sigma-Aldrich Merck (Johannesburg, South Africa). To determine the selectivity of the method in the separation of the targeted flavonoids, 100 ppm stock solutions of the pure standards (apigenin, apigetrin, luteolin, luteoloside, naringenin, vicenin-2, vicenin-3, vitexin, isovitexin, and internal standard D-fluorophenylalanine (Table S3)) were separated using reverse phase chromatography followed by multiple reaction monitoring (MRM) quantification [21]. The prepared stock standard solutions were analyzed using a C18 reverse phase chromatography column (Restek AQ, 100 mm × 2.1 mm × 3 μm) on a Shimadzu Nexera 20A UHPLC system connected to a Shimadzu 8050 triple quadrupole mass spectrometer with an ESI interface switching between both positive and negative ionization modes (Shimadzu, Kyoto, Japan). The standards were injected in triplicate and separated using a binary gradient (Solvent A: Pure MilliQ Water/ 0.1% (*v*/*v*) formic acid, Solvent B: UHPLC grade methanol/ 0.1% (*v*/*v*) formic acid) at a 0.40 mL/min flow rate. In the binary gradient, the concentration of solvent B was increased in 5% (*v*/*v*) increments to 25% (*v*/*v*) at 2–18 min and 95% (*v*/*v*) at 25–30 min before reducing the concentration to 2% at 31 min. 

The instrumental conditions for MS/MS detection performed in positive and negative ion modes with MRM scanning were set as follows: 3 L/min nebulizing gas flow, 15 L/min drying gas flow, 4.5 kV interface voltage, 400 °C heat block temperature, 250 °C desolvation temperature, 1.6 × 10^−3^ Pa ion gauge vacuum. The precursor ions were directly infused into the triple quadrupole MS in the multiple reaction monitoring mode to determine the optimum conditions for generating product ions to be used for quantification. Briefly, the individual precursor ions were selected in the first quadrupole (Q1), followed by collision-induced dissociation (Q2) with nitrogen gas at 230 kPa, and transitioned into product ions, which were detected in the third quadrupole (Q3). The built-in (vendor specific) LabSolutions optimization software (Shimadzu, Kyoto, Japan) optimized the individual collision energies (CEs) for the authentic standard compounds. The most favorable ESI mode was determined from the peaks with the highest intensity. The optimized CE, precursor-to-product ion transitions, and product ions of the individual standard compounds are summarized in Table S3. 

For the preparation of calibration curves, standard stock solutions of 100 ppm were prepared from the pure standard compounds dissolved in a 50% (*v*/*v*) UHPLC Grade Methanol/MilliQ Water solvent. Serial dilutions were prepared to 5, 1, 0.5, 0.25, 0.1, and 0.05 parts per million (ppm, 1 ppm = 1 mg/L). The standards were prepared in triplicate and analyzed as mentioned above, with each sample injected in triplicate. The standard curves were constructed by plotting the acquired integrated peak area against the standard concentration. The standard curve equations and R^2^ values are summarized in Table S4. The obtained values in ppm were converted to ng/g plant leaf material (wet weight).

The limit of detection (LOD), the lowest analyte concentration that can be detected with a 1:10 signal-to-noise (S/N) ratio, and the limit of quantification (LOQ), the lowest level at which the analyte can be quantified with a 1:3 signal-to-noise (S/N) ratio [21], were determined by the preparation of serial dilutions (0.00025–5 ppm) of the pure standard solutions to determine the lowest detectable concentrations, as analyzed on the UHPLC-3Q-MS system. The LOD and LOQ of all the standard compounds and internal standard were determined at 0.025 ppm and 0.05 ppm, respectively.

SPSS software (IBM SPSS Statistics, version 29 (IBM Corp., Armonk, NY, USA) was used for descriptive statistics. Here, one-way analysis of variance (ANOVA) was performed to compare the mean values of individual metabolites at different time points. ANOVA was followed by the Tukey post hoc test, where differences between the means were considered significant at *p* < 0.05, indicated in graphs with an asterisk.

## 3. Results and Discussion

Early development is a perilous stage in the life of seedlings, as the plant must establish itself in the environment during this time. As a strategy to contribute to survival, plants utilize the synthesis of specialized metabolites during this phase [22], which involves interactions with beneficial or pathogenic microorganisms and the deployment of possible defense mechanisms [23,24]. Thus, the focus was to interrogate the metabolomic reprogramming of sorghum seedlings over the early growth period following germination to identify key metabolite markers that define the early development of the plant.

### 3.1. UHPLC-MS Analyses of Sorghum Leaf Extracts and Initial Data Analysis

The methanolic extracts from different developmental stages displayed inherent multidimensionality due to the complex physicochemical characteristics (e.g., polar vs. nonpolar; aglycone vs. glycosides) of the sample constituents. The UHPLC-MS chromatograms revealed differential profiles, which included variation in peak intensities and presence/absence of peaks. This indicates differential metabolite composition and content and is indicative of time-related changes to the metabolomic architecture (Figure S2A,B). 

Untargeted metabolomics generates very large datasets that can be approached using two methods: (i) the traditional route, whereby the initial steps entail the application of chemometrics methods, followed by the annotation of the selected discriminant variables, and (ii) targeted profiling, whereby metabolite annotation is initially performed, then followed by the application of chemometrics methods [25,26,27]. The latter route was followed in this study, with the focus on previously identified key metabolites in the defense response of sorghum seedlings [9,10,14].

**Figure 1:**
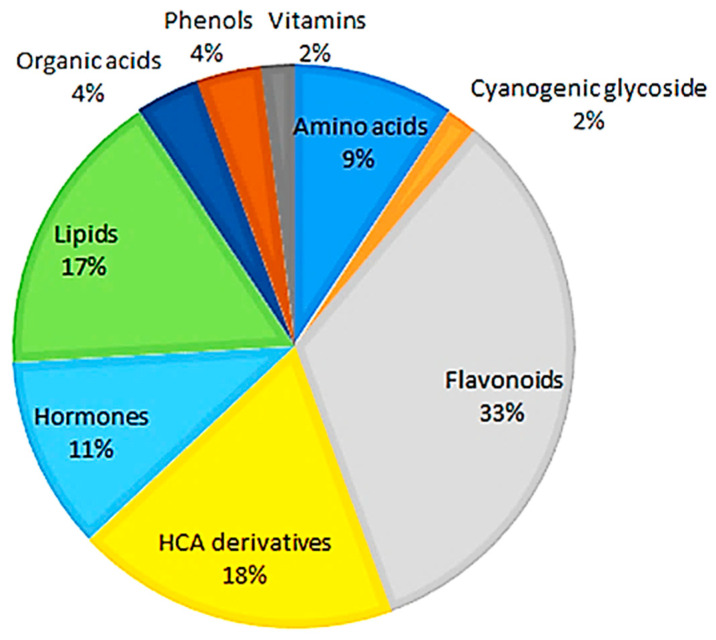
Classes of annotated metabolites present in hydromethanolic extracts of developing *Sorghum bicolor* seedlings. Of all the annotated metabolites (Table S1), the flavonoids, derivatives of hydroxycinnamic acids, and lipids were the major classes associated with early growth and development across all time points, days 7–29.

The metabolites are of different chemical classes, including amino acids, cyanogenic glycosides, flavonoids, hydroxycinnamic acid (HCA) derivatives, hormones, lipids, organic acids, and other phenolics (Table S1). As shown in Figure 1, the flavonoid class of phytochemicals forms the largest group of metabolites, while the cyanogenic glucoside class forms the smallest group compared to all of the other classes of metabolites. 

**Figure 2:**
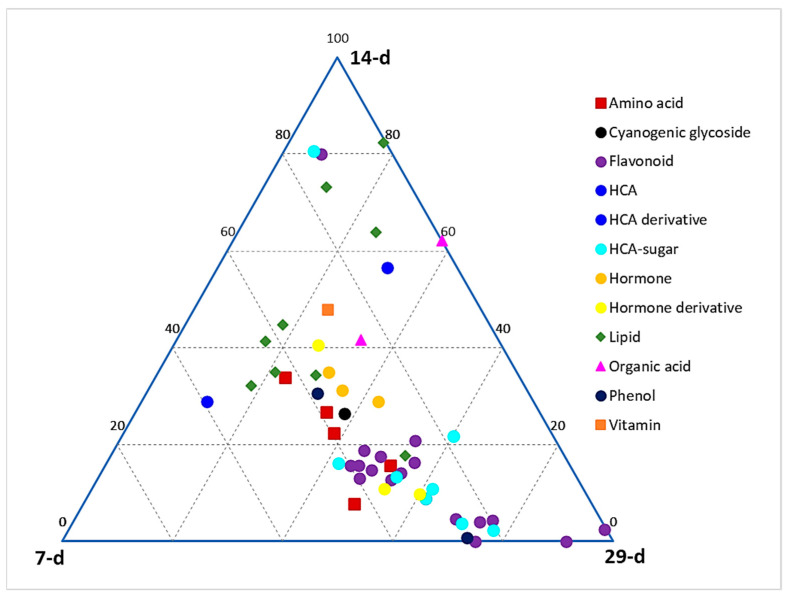
Ternary plot showing all classes of annotated metabolites present in hydromethanolic extracts of developing *Sorghum bicolor* seedlings. Seedlings were harvested at early (7 d), middle (14 d), and late (29 d) growth stages. The plot was constructed based on annotated metabolites across all time points, as listed in Table S1.

A ternary plot (Figure 2) was generated to depict the global relative quantification levels of different classes of metabolites across the early, middle, and late seedling developmental stages (corresponding to days 7, 14, and 29 post-germination, respectively). This plot shows the relative quantification of members of each metabolite class at each of the three stages, as represented on the ternary at each point. At each apex of the plot is the maximum relative quantification of the metabolites at that particular growth stage (i.e., 100%), which then decreases along the axes towards the next apex. It allows us to see how the points cover the space and to detect potential zones that should be explored in further experiments. From a global point of view, a larger content of metabolites is observed at the late stage as compared to the middle and early stages. It can also be deduced that the phenylpropanoid-derived phenolic compounds (HCA derivatives and flavonoids) are more prevalent in the late stage compared to early and middle stages, whereas lipids are more concentrated in the early and middle stages compared to the late stage. Other classes, such as amino acids and hormones, are clustered between the early and late stages. 

### 3.2. Multivariate Data Analysis and Chemometric Modelling 

**Figure 3:**
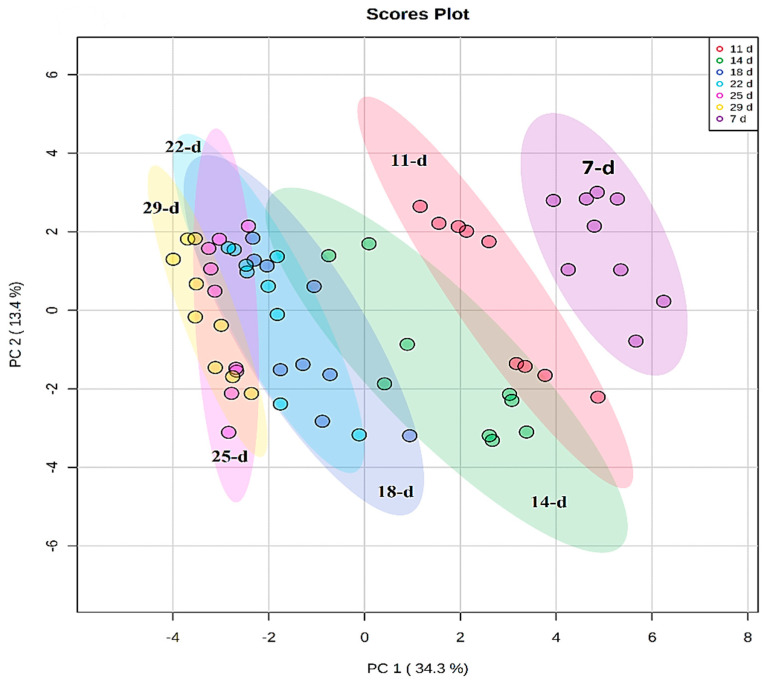
Unsupervised chemometric analysis of annotated metabolites present in extracts from *Sorghum bicolor* seedlings at different developmental stages. Principal component analysis (PCA) plots were constructed using MetaboAnalyst: A scores scatter PCA plot (PC1 vs. PC2) of log-transformed and Pareto-scaled MS data from seedlings on days 11, 14, 18, 22, 25, and 29 post-germination. The clusters are colored based on the different developmental stages (purple = 7 d, red = 11 d, green = 14 d, blue = 18 d, turquoise = 22 d, pink = 25 d, and yellow = 29 d). The PC analysis generated 9 principal components, of which 64.9% of the total component variation was captured.

Chemometric modelling was applied to 46 annotated metabolites that exhibited variation in peak intensities (Table S1) to understand and describe the developmental changes through the lenses of the annotated metabolic profiles. PCA modelling was performed to observe the natural structures in the dataset. PCA is an unsupervised method that depicts similarities and differences (intrinsic interconnectedness) between samples within a dataset by reducing the dimension or complexity of the data, thus allowing for an interpretable visualization and analysis. As such, the PCA methods provide a qualitative representation of the similarities and differences (variation) between and within the samples [28]. The first two principal components (PC1 and PC2) of the generated PCA model explained 47.7% of the total variation and revealed a time-related trend, which reflects the differential metabolome changes across the developmental stages (7, 11, 14, 18, 22, 25, and 29 d) of the seedlings (Figure 3). 

**Figure 4:**
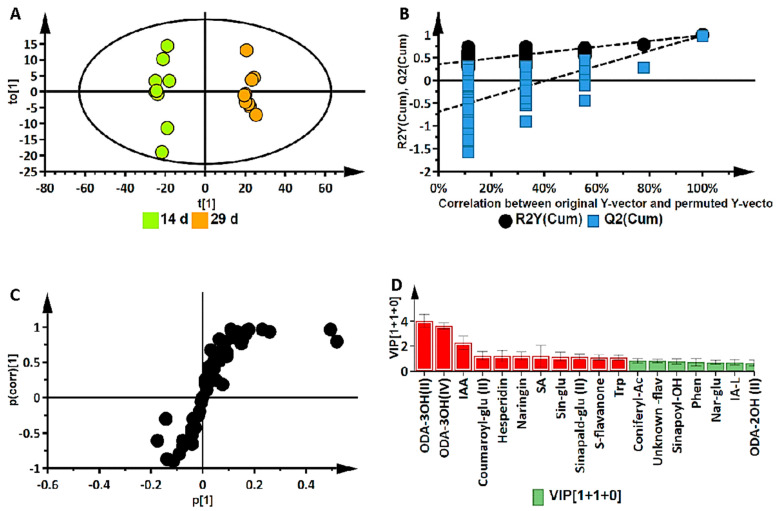
Supervised multivariate data analysis of annotated metabolites in *Sorghum bicolor* seedlings following UHPLC-MS analysis of the 14 d vs. 29 d group samples. (**A**) An OPLS-DA scores plot of the predictive component t[1] and the first orthogonal component t0[1], R^2^(cum): 0.989; Q^2^(cum): 0.980; R^2^ permutation: 0.259; Q^2^ permutation: −0.541; *p*-value: 0.0000; and components: (1+1+0). The ellipse indicates the 95% limit of the Hotelling T^2^ distribution for the model. (**B**) Permutation analysis plotting R^2^ and Q^2^ (black and blue dots, respectively) from n = 100 permutation tests in the OPLS-DA model. The y-axis shows R^2^ and Q^2^, whereas the x-axis shows the correlation coefficient of permuted and observed data. The cluster of points on the left represents 100 permuted R^2^s and Q^2^s, and the two points on the right represent the observed R^2^(cum) and Q^2^(cum). Dashed lines denote corresponding fitted regression lines for observed and permutated R^2^ and Q^2^. (**C**) OPLS-DA S plot with discriminant biomarkers at each end of the S-plot. The covariance (variable magnitude) and correlation (reliability) of the variables in the model (indicated with black dots) are represented on the axes as p[1] and p(corr)[1], respectively. The features located at the extreme ends of the plot show a positive association (high magnitude and high reliability) to the respective conditions being compared, while those in the middle can be regarded as shared features. (**D**) A VIP plot where the metabolites of a VIP score of >1 is significant to metabolomic differences corresponding to developmental changes. (Metabolite abbreviations: ODA-3OH(II) = trihydroxy-octadecadienoic acid; ODA-3OH(IV) = 9,12,13-trihydroxy-10-octadecenoic acid; IAA = indole-3-acrylic acid; coumaroyl-glu II = coumaroyl glucose; SA = salicylic acid; sin-glu = sinapaldehyde glucoside I; sinapald-glu II = sinapaldehyde glucoside II; S-flavanone = sophoraflavanone; trp = tryptophan; coniferyl-Ac = coniferyl acetate; sinapoyl-OH = sinapoyl-alcohol; phe = phenylalanine; nar-glu = naringenin 7-O-beta-D-glucoside; IA-L = indole-3-acetyl-leucine; ODA-2OH (II) = 9,10-dihydroxy-12-octadecenoic acid).

While the ternary plot and PCA provided a global view of the metabolome changes in terms of metabolite classes at different developmental stages, orthogonal projection to latent structures discriminant analysis (OPLS-DA) was applied to 43 annotated metabolites for the binary classification of samples to further investigate these changes. OPLS-DA performs sample classification based on linear regression, where differences among groups are modelled; it also identifies discriminant molecules specific to those groups. Supervised methods use predictive models to identify biological responses relating to certain variables, thereby identifying independent and dependent variables in a dataset. The calculated OPLS-DA models were thus computed to separate multivariate relationships into predictive variation and orthogonal variation. The binary classification of the seedlings at the 14 d stage and the 29 d stage, showing the clear separation of the two groups, is represented by the OPLS-DA scores plot in Figure 4A (14 d vs. 29 d). The corresponding figures for 7 d vs. 14 d and 7 d vs. 29 d are presented as Figure S3A,B, respectively.

The OPLS-DA models were validated by diagnostic statistics to ensure the reliability of the models and to prevent the over-fitting of the models to the data. As indicated in the experimental section, the cross-validation (CV) method and permutation tests were used in this research [15]. The permutation test performed was with 100 iterations (*n* = 100). Permutation tests, as shown in Figure 4B, are randomization-based validation methods that are employed to validate the predictive power of OPLS-DA models, comparing the R^2^ and the Q^2^ of both the permuted and original models. 

The significant metabolites characterizing the early, middle, and late (corresponding to days 7, 14, and 29) development stages were then selected using OPLS-DA S-plots in Figure 4C and Figure S4A,B. These loading S-plots identify the variables that contribute the most to the pattern changes observed on the OPLS-DA scores plots. The variables with the highest absolute values of p[1] and p(corr)[1] are those identified as the discriminant markers and these express/represent the differences between the different groups [29].

The *p*-value obtained from the OPLS-DA model in Figure 4A was 0.0000, determined by the CV-ANOVA (analysis of variance of cross-validated parameters) test, which indicates that there is a statistically significant difference between the two groups and that the null hypothesis (that there are no differences between the groups) can be rejected. In addition, a VIP plot metric was used for validation of selected metabolites from the S-plots. With VIP, metabolites are scored as a measure of how much they contribute to the model, and the variables that are of significance are those with a VIP score > 1. A higher VIP score is directly related to the significance of a variable. The VIP plot in Figure 4D shows the top 18 most significant metabolites, differentially expressed in the metabolomes of the 14 d and 29 d stages of seedling growth. The metabolites highlighted in red are those correlating to the late (29 d) stage, while the rest are those corresponding to the mid (14 d) stage.

### 3.3. Deriving Biochemical Insights from Metabolomics Data

Hierarchical cluster analysis (HiCA) was applied to further explore the time-related trends and groupings revealed by the PC analysis. The HiCA shows the statistical correlation amongst all the samples analyzed and then builds a hierarchy from them. Consequently, similar samples consisting of similar metabolomes form clusters together, generating groups formed on the basis of cluster similarity [30]. Furthermore, the HiCA heatmap also shows the relative abundance of the identified metabolites, thereby displaying the variation of metabolic features present at the different developmental stages of the seedlings.

**Figure 5:**
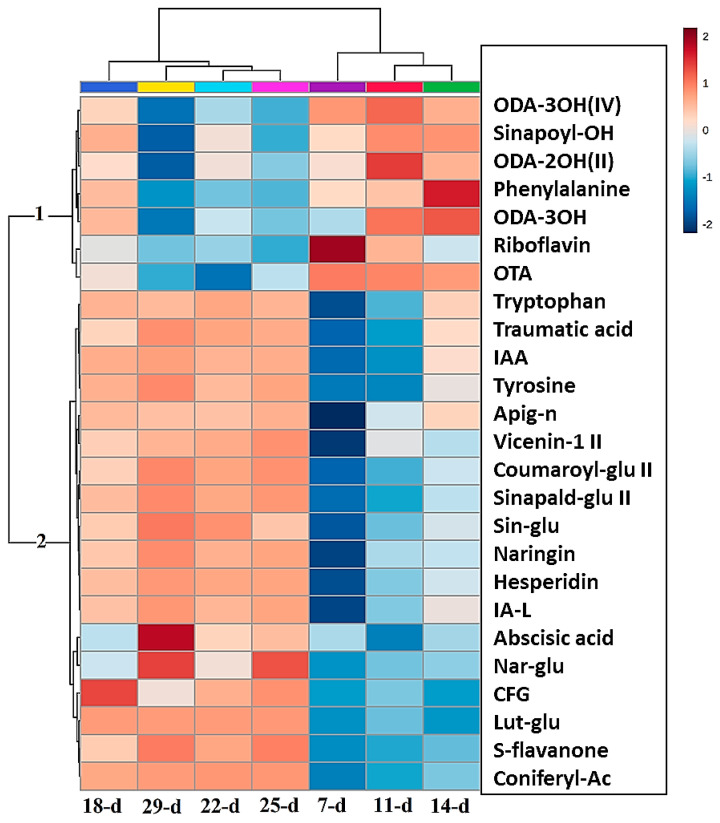
Hierarchical cluster analysis (HiCA) heat map of the top 25 metabolites present in the averaged extracts (*n* = 9) showing the most and least abundant metabolites at each developmental stage (red/blue = high/low concentrations, respectively). The metabolites listed are as follows, respectively: 9,12,13-trihydroxy-10-octadecenoic acid (ODA-3OH(IV)), sinapoyl alcohol (sinapoyl-OH), 9,14-dihydroxy-10,12-octadecadienoic acid (ODA-2OH(II)), phenylalanine, trihydroxy-octadecadienoic acid (ODA-3OH(II)), riboflavin, octadecatetraenoic acid (OTA), tryptophan, traumatic acid, indole-3-acrylic acid, tyrosine, apigenin 7-O-neohesperidoside (rhoifolin) (apig-n), apigenin 6-C-xyloside-8-C-glucoside (vicenin-1), coumaroyl glucose (coumaroyl-glu II), sinapaldehyde glucoside II (sinapald-glu II), sinapaldehyde glucoside I (sin-glu), naringenin 7-O-neohesperidoside (naringin), hesperidin, indole-3-acetyl-leucine (IA-L), abscisic acid (ABA), naringenin 7-O-beta-D-glucoside (nar-glu), 1,3-O-coumaroyl-feruloylglycerol (CFG), luteolin 7-O-glucoside (Lut-glu), sophoraflavanone G (S-flavanone), coniferyl acetate (coniferyl-Ac).

This HiCA heatmap (Figure 5) shows the average levels of each of the top 25 metabolites at each developmental stage. These patterns suggest that differential reprogramming has occurred over time. This can take the form of high or low accumulation at specified time points, indicating early, late, or oscillatory responses. Where molecules show increasing or decreasing trend patterns, it could be suggestive of increased biosynthesis followed by interconversion, conjugation, degradation, or incorporation into insoluble polymers such as lignin [23,31].

Two significant groups (1 and 2) are observed in the map that display different patterns of metabolite abundance at each of the time points. The metabolites in group 1 show increased levels in the early stages of development (days 7 to 14) compared to the late stages (days 18 to 29), where the levels of these metabolites decreased. Conversely, the metabolites in group 2 show a linear increase in their levels as the seedlings progress in growth from the early stages to the late stages of development, i.e., from days 7 to 29. The metabolites of group 1 are mostly lipids (ODA-2OH II, ODA-3OH, ODA-3OH IV, and OTA; abbreviations are defined in Figure 5). The other metabolites in this group are phenylalanine, sinapoyl alcohol, and riboflavin, which are from the amino acid, HCA, and vitamin classes, respectively. Significant amounts of lipids are stored in the seeds of plants, that are activated after seed germination. This aids in the growth and development of the seedlings before photosynthesis is initiated. Most of these lipids, such as octadecatetraenoic acid (OTA), act as signalling molecules in plant defense systems [32]; therefore, they are at high levels in the early stages of plant growth, as shown in Figure 5. In contrast, group 2 contains mostly flavonoids. These are not essential for the early survival of seedlings, but more important for the phototrophic response of the seedlings when photosynthesis is initiated; therefore, these metabolites are at low levels in the early growth stages and at high levels in the late stages [33].

The discriminant metabolites identified by the OPLS-DA S-plots that were positively correlated with the 14 d stage were phenylalanine, sinapoyl alcohol, 9,14-dihydroxy-10,12-octadecadienoic acid (ODA-2OH(II)), trihydroxy-octadecadienoic acid (ODA-3OH(II)), and 9,12,13-trihydroxy-10-octadecenoic acid (ODA-3OH(IV)). From the same OPLS-DA S-plot, coniferyl-acetate, 1-O-coumaroyl-beta-D-glucose, sinapaldehyde glucoside, sophoraflavanone G, naringenin 7-O-neohesperidoside, an uncharacterized flavonoid, hesperidin, indole-3-acrylic acid, and tryptophan showed a positive correlation with the 29 d stage. In the growth condition of 7 d vs. 14 d, as shown in Figure S4A, isocitric acid, 15-hydroxylinoleic acid, 9,12,13-trihydroxy-10-octadecenoic acid, and riboflavin showed a positive correlation with the 7 d stage. In the 7 d vs. 29 d growth stage condition, depicted in Figure S4B, the metabolites that were positively correlated to the 7 d stage were trihydroxy-octadecadienoic acid, 15-hydroxylinoleic acid, 9,12,13-trihydroxy-10-octadecenoic acid, and riboflavin, which is similar for the 7 d vs. 14 d condition.

### 3.4. Pathway Enrichment Analysis Indicates Importance of the Phenylpropanoid Pathway

Analysis of metabolic pathways potentially provides insight into the most significant biochemical and physiological processes occurring in the developing seedlings. These pathways are recognized by identifying groups of metabolites belonging to the same metabolic network.

The KEGG IDs of each compound were used to map these metabolites into principal metabolic pathways. The most significant primary pathways identified by this approach, (listed in Table S2) include phenylalanine, tyrosine, and tryptophan biosynthesis, riboflavin metabolism, and glyoxylate and dicarboxylate metabolism. The most significant pathways identified from the analysis include isoquinoline alkaloid biosynthesis, phenylalanine metabolism, phenylpropanoid biosynthesis, and flavone and flavonoid biosynthesis. Flavone and flavonol biosynthesis had the most hits when arranged by the pathway impact (≥0.1) and when arranged by FDR (≤0.5); phenylpropanoid biosynthesis had the most hits. This was followed by aromatic amino acid biosynthesis (Trp, Tyr, and Phe, with Phe feeding into the phenylpropanoid pathway). Some of the most abundant specialized metabolites in plants originate from these aromatic amino acids.

Biosynthesis of specialized metabolites is based on the formation of precursors of specialized metabolites produced by primary metabolism [34]. These precursors are mostly produced from glycolysis, the tricarboxylic acid (TCA) cycle, etc. Isoquinoline alkaloid biosynthesis produces precursors of alkaloids by decarboxylation of tyrosine. Similarly, they can also be derived from the amino acids Phe, Lys, Trp, and Tyr. The precursor compound, 4-coumaroyl CoA, which supports the production of flavonoids, is itself produced in the phenylpropanoid pathway. Intermediates emerging from the shikimate pathway act as precursors for the phenylpropanoid pathway. Together, most of the sorghum specialized metabolites are derived from these two pathways [35].

Pathway enrichment analysis is based on the quantitative information of the statistically significant compounds and contributes to reducing the complexity of the metabolomes. Its application identified enriched pathways in the developing seedlings and compared pathway functionalities at the different stages when growth is rapid and associated with dynamic changes. Furthermore, pathway topology analysis was applied to identify the connections between the metabolites within the metabolic pathways [19].

**Figure 6:**
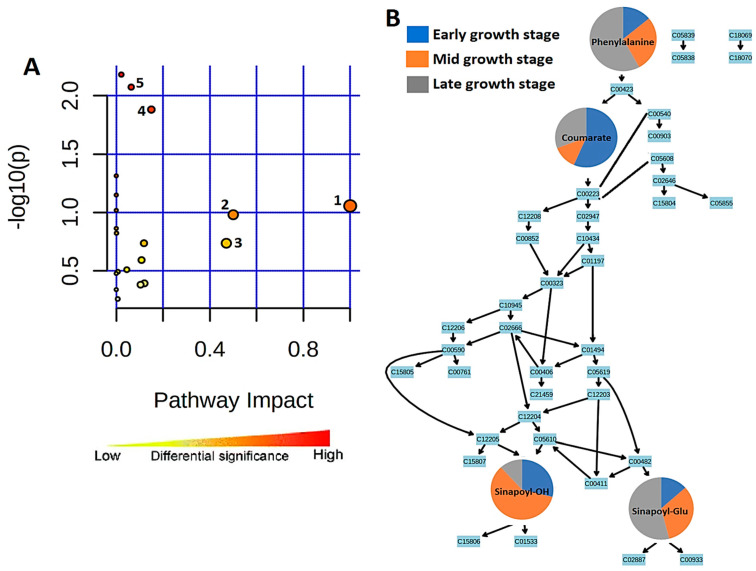
Summarized representation of metabolic pathway analysis. (**A**) Pathways are arranged by increasing pathway impact, which represents differential significance, on the x-axis and by increasing *p*-value on the y-axis. Each pathway is represented by a circle in which size/radius increases with increasing impact, and the color of the circle also becomes more red as the *p*-value increases. 1, Biosynthesis of specialized metabolites. 2, Isoquinoline alkaloid biosynthesis. 3, Phenylalanine metabolism. 4, Flavone and flavonoid biosynthesis. 5, Phenylpropanoid biosynthesis. (**B**) A representation of the topological properties of the phenylpropanoid pathway. The changes in the four matched metabolites in their early, middle, and late seedling growth stages are represented by pie charts, indicating stage-specific quantitative variation.

The most significant metabolic pathways are represented by the pathway analysis in Figure 6A. Topologically, the phenylpropanoid biosynthesis metabolic pathway is represented in Figure 6B, where four of the metabolites annotated from the OPLS-DA data and matched to the pathway are found. These are phenylalanine, 4-coumarate, sinapoyl alcohol, and sinapaldehyde glucoside.

In the early stage of sorghum seedlings (day 7), phenylalanine is present at low levels. This could be due to the amino acid being converted to 4-coumarate at the start of the pathway. However, phenylalanine accumulates over the period of investigation to day 29, reflecting the increase in flavonoid synthesis and the demand for lignin precursors associated with cell wall synthesis. Coumarate then gives rise to sinapoyl alcohol and sinapaldehyde glucoside further down the metabolic pathway. Sinapoyl alcohol is highly abundant in the middle 14 d stage of seedling growth and is at its lowest level in the late stage. However, sinapylaldehyde glucoside is at its highest level in the late growth stage and is lowest in the early stage, indicative of the glycosylation of the former to the latter.

### 3.5. Relative Quantification of Selected Discriminant Metabolites

**Figure 7:**
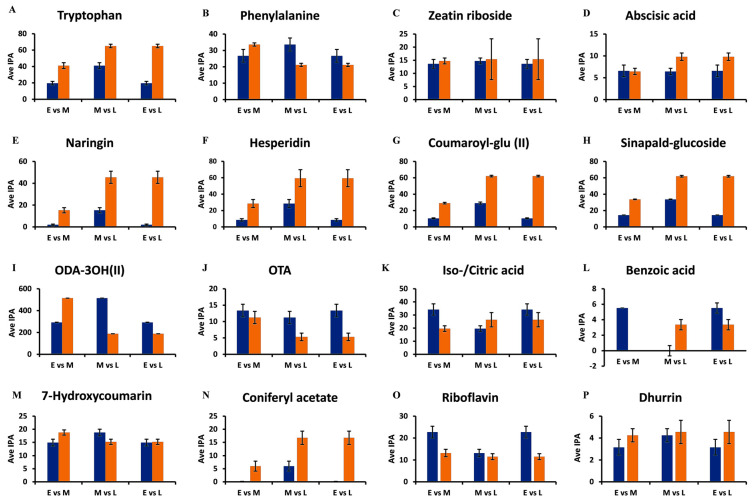
Representative bar graphs showing the semi-quantification of discriminant metabolites retrieved from the OPLS-DA S plots. The average integrated peak area values (y-axis, Ave IPA, n = 9) of metabolites in the early (E = 7 d), middle (M = 14 d), and late (L = 29 d) growth stages are compared to illustrate developing trends within the metabolomes. In the paired comparisons, blue indicates the earlier time point (E or M), while orange indicates the later time point (M or L). (**A**,**B**) Tryptophan and phenylalanine of the amino acid class. (**C**,**D**) Zeatin riboside and abscisic acid of the hormone class. (**E**,**F**) Naringenin 7-O-neohesperidoside (naringin) and hesperidin of the flavonoid class. (**G**,**H**) Coumaroyl glucose and sinapylaldehyde glucoside of the HCA derivatives. (**I**,**J**) Trihydroxy-octadecadienoic acid and octadecatetraenoic acid of the lipid class. (**K**,**L**) Iso-/citric acid and benzoic acid of the organic acid class. (**M**,**N**) 7-Hydroxycoumarin and coniferyl acetate of the phenol class. (**O**) Riboflavin of the vitamin class and (**P**) Dhurrin of the cyanogenic acid class. The error bars indicate the standard deviation.

In order to gain insights into the dynamics of the changes occurring during the investigated growth period, relative quantification was performed on the selected discriminant metabolites. The bar graphs in Figure 7 show the changes in the levels of the different metabolites in the seedlings, as determined at the 7 d, 14 d, and 29 d stages.

The levels of tryptophan increase from the early 7 d stage, through to the middle 14 d stage, and finally to the late 29 d growth stage (Figure 7A). Important specialized plant metabolites are derived from tryptophan [36], such as alkaloids and indole-3-acetic acid (IAA), which contribute to plant defense. Therefore, as the seedlings grow, defensive metabolites also increase in concentration [36]. Phenylalanine, on the other hand, increased from the 7 d stage and was at its highest concentration at the 14 d stage, followed by a decrease at the 29 d stage (Figure 7B).

Phenylalanine connects primary and secondary metabolism in plants through the induced action of phenylalanine ammonia lyase (PAL), in response to developmental cues and stress triggers. PAL generates trans-cinnamic acid, which feeds into the early phenylpropanoid pathway, which functions in plant defense and structural support through the synthesis of lignin precursors [37]. In turn, phytohormones, such as abscisic acid and zeatin riboside function in plant growth and plant responses to environmental stress [38]. It was observed that the concentrations of both of these compounds increased from the early 7 d stage to the late 29 d stage of the seedlings (Figure 7C,D).

Plant phenolic compounds (e.g., HCAs, hydroxybenzoic acids, and flavonoids) often exhibit antioxidant properties that allow for adaptation to changing environmental conditions [39]. However, compounds that contain phenolic groups might be active as antimicrobial agents as well, causing a notable correlation between the total phenolic content and antioxidant activity [6,40]. Moreover, some overlap/correlation between antioxidant activity and antimicrobial activity might be found [6,39]. These effects might act synergistically to create an enhanced antimicrobial environment in planta. Unfortunately, little information is available regarding sorghum seedlings [7].

Two important sub-classes of phenolics are the flavonoids and the HCA derivatives. Flavonoids form the largest class of specialized metabolites in most plants and perform several critical functions related to development and environmental adaptation, including plant–microbe interactions [41,42]. Both naringenin 7-O-neohesperidoside (naringin) and hesperidin increased as the seedling progressed in growth from the 7 d stage to the 29 d stage (Figure 7E,F). HCA derivatives and conjugates are compounds derived from core phenylpropanoids that act as antioxidants [43]. In addition, members of this class display antimicrobial activity. In plants, their major role is to provide a measure of defense and thus resistance against pathogens. This property is essential to vulnerable plants to allow seedlings to adapt to the environment in their early stages of growth and development. The HCA derivatives, coumaroyl glucose, and sinapylaldehyde glucoside (Figure 7G,H) similarly display the same pattern of increasing concentrations from the early to the late growth stages. These increases are due to the biosynthesis of HCAs in the early phenylpropanoid pathway, which are precursors for various mono-lignols. The compounds contribute to the lignification of cell walls of newly formed cells, thereby supporting the progressive growth and development of plants and strengthening the cell walls, providing plant defense [44].

Fatty acids are essential members of the lipidome and lipid metabolism and contribute to the synthesis of cell membranes with the associated structural and functional properties. Moreover, they can act as signalling molecules or lipid mediators in plants [32,45]. The oxygenated fatty acids, trihydroxy-octadecadienoic acid and octadecatetraenoic acid in Figure 7I,J, mostly act as signalling molecules [46]. The concentration of trihydroxy-octadecadienoic acid increases from 7 d to 14 d, then decreases at the 29 d growth stage. Octadecatetraenoic acid decreases in its concentration from the 7 d stage to the 29 d stage.

Organic acids contribute to carbon metabolism in plants and take part in the biochemical pathways in plant cells, such as the TCA cycle [47]. Isocitric acid decreases in its levels from the early growth stage to the late growth stage, as depicted by Figure 7K,L, with 14 d having the lowest level of isocitric acid metabolites. Together with the amino acid data, these changes might reflect C and N metabolism and link the related metabolic cycles of anaplerotic reactions to counteract depletion of the TCA cycle by biosynthetic demands. Relatedly, shifts in carboxylic acids levels were reported to be perceived in plants during stress and it was proposed that the tricarboxylates could modulate signal transduction events linked to plant defense [48].

The benzoic acid levels are almost absent at the 14 d stage but are at their highest level at the 7 d stage, decreasing at the 29 d stage. The metabolic changes for the phenolic metabolites 7-hydroxycoumarin and coniferyl acetate are depicted in Figure 7M,N. According to the figures, 7-hydroxycoumarin (umbelliferone) shows minor fluctuations around the middle growth stage. In contrast, coniferyl acetate is at extremely low levels at the 7 d stage but increases at the late 29 d growth stage.

The lowest abundant metabolites are riboflavin and dhurrin, a cyanogenic acid, shown in Figure 7O,P. Riboflavin, a cofactor for diverse metabolic processes and inducer of plant resistance, decreases from the 7 d stage to the 29 d stage. Conversely, durrhin, which is a wound metabolite and source of resistance of sorghum seedlings to fungal infection [49], is already present at the 7 d stage and increased in concentration at the 29 d stage.

### 3.6. Quantitative Determination of Flavones

As a phytochemical class, flavonoids are composed of flavonols, flavanones, flavanols, flavones, isoflavones, and their respective derivatives [42]. Flavone O-conjugates and their aglycones, (e.g., apigenin and luteolin) are constitutive metabolites in stem and leaf tissues of grasses. Apigenin and luteolin are generally O-glycosylated; however, flavone C-glycosides, which contain sugar residues covalently linked to C-6 and/or C-8 in the flavonoid A-ring, co-exist with flavone O-conjugates as predominant flavonoids [33,50].

In particular, flavonoids are considered as plant UV protectants due to their epidermal accumulation and radical scavenging properties [35,42]. In grasses, accumulation of flavone aglycones and their O-conjugates in grasses can be triggered by biotic stresses. For example, apigenin and luteolin are defense-related metabolites in sorghum infected by the anthracnose fungus *Colletotrichum sublineola* [10,51]. These pathogen-inducible flavones inhibit the in vitro spore germination of *C. sublineola*, implicating a functional role in chemical defense [51]. In addition, apigenin and luteolin and their O- and C-glycosylated derivatives have been reported to possess direct antimicrobial activity [8,52] and were also found as discriminant metabolites in sorghum seedlings responding to *Burkholderia andropogonis* [9] and *C. sublineola* [10].

**Figure 8:**
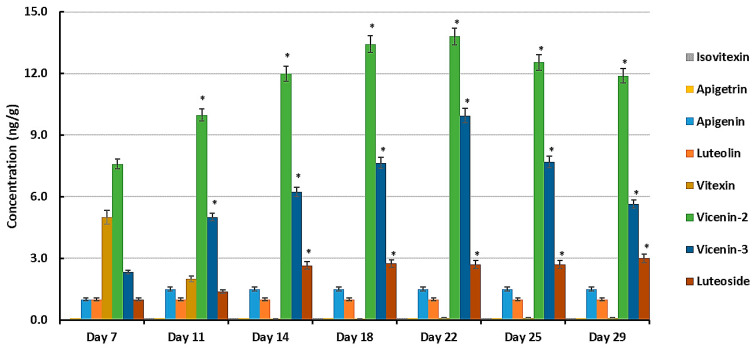
The varying concentrations of flavones apigenin and luteolin and the glycosylated derivatives vitexin, vicenin-2, and vicenin-3 and luteoside in developing seedlings of the sorghum cultivar NS5511 at 7, 11, 14, 18, 22, 25, and 29 days post-germination, as determined by UHPLC-3Q-MS/MS using optimized MRM conditions. All concentrations (y-axis, ng/g fresh weight) are mean concentration values with *n* = 9, and error bars indicate the standard error. Isovitexin and apigetrin were below the limit of detection. An asterisk (*) indicates the statistical significance (ANOVA followed by Tukey post hoc test at a *p*-value < 0.05 when comparing mean values to that of day 7).

In addition to other flavonoids, Table S1 lists the following derivatives of two flavones, apigenin and luteolin (3′-hydroxyapigenin), as discriminant metabolites: apigenin 8-C-glucoside (vitexin), apigenin 7-O-glucoside (apigetrin), apigenin 6-C-glucosyl-8-xyloside (vicenin-3), apigenin 6-C-xylosyl-8-C-glucoside (vicenin-1), apigenin 7-O-neohesperidoside (rhoifolin), luteolin 7-O-glucoside (luteoloside), luteolin 7-O-neohesperidoside (lonicerin), with the flavanone naringenin as precursor (naringenin 7-O-beta-D-glucoside and naringenin 7-O-neohesperidoside or naringin) (Figure S5). Quantitative studies of selected flavonoids can play a significant role in deciphering data acquired from untargeted and targeted metabolomic studies. Their concentrations were thus further investigated using quantitative MRM analysis during the same time periods as used for the metabolomics analysis [9]. The mean peak areas from the UHPLC-MS/MS data along with the respective standard curve equations were used to determine the concentration values in ng/g (Figure 8).

The flavones luteolin, luteoloside, vicenin-2, vicenin-3, and vitexin were detected at varying concentrations at different d.p.g., but apigetrin and isovitexin were below the LOD. From day 11, the dominant flavones were vicenin-2, vicenin-3 (with apigenin as aglycone), and to a lesser extent, luteoside (with luteolin as aglycone). Vicenin-2 and vicenin-3 were the most abundant at all stages of development, which may suggest that they play a significant role in the preparation of the plants’ defense against stresses during early development. Of interest is the profile of vitexin, which is initially relatively high (day 7, decreasing to day 11), followed by its apparent disappearance from the samples (day 14 onwards). Vitexin is known to occur in sorghum seeds [53] and it is possible that some of the metabolite could have originated from the germinating seed. Vitexin is an 8-C-mono-glycosylated form of apigenin that is metabolized to the diglycosylated vicenins (Figure S5). The consistent low level of apigenin can be explained in view of its role as precursor compound (aglycone) of the vicenins, thus expanding the multi-metabolite apigenin-based chemical space in the seedlings. A similar situation my apply in the case of luteolin and luteoloside.

Previous investigations on metabolomic profiling of the response of sorghum seedlings to pathogen attack have revealed the reprogramming of pathways that synthesize flavonoids (especially flavones) in the sorghum metabolome post-infection with *B. andropogonis* and *C. sublineola*, respectively [9,10]. The flavones upregulated in both pathogen responses were identified as apigetrin, apigenin, vitexin, isovitexin, luteolin, luteoloside, vicenin-2, and vicenin-3, indicating a positive correlation of the glycosylated derivatives of apigenin and luteolin with the defense response of sorghum seedlings [54]. Although glycosylated flavonoids appear to have a reduced antimicrobial effect compared to the respective aglycones [55], they can serve as storage forms of constitutive anti-microbial metabolites for the rapid release of the aglycones upon pathogen attack.

## 4. Conclusions

The seedling stage of plant development is the most crucial and vulnerable to varying environmental conditions. Various metabolic pathways occur within sorghum seedlings to support biological functions essential to the growth and development of the plants as well as to support innate immune responses in defense against attempted pathogen attacks. The study of the development-related metabolomes provided superior insights into these processes as the seedlings progressed in their initial post-germination growth. Changes in the metabolomes, as evident from PCA plots, showed distinct clustering. Significant changes in the metabolic patterns of these metabolomes were focused in the early, middle, and late growth stages of the seedlings. In turn, OPLS-DA was utilized to identify discriminatory markers associated with specific developmental stages. Nine classes of specialized metabolites associated with the changing metabolomes were identified from the annotated UHPLC-MS datasets.

Pathway analysis revealed five metabolic pathways, with the phenylpropanoid pathway being the most pronounced and flavonoids the most abundant class of metabolites present in the samples. Flavonoids and HCA derivatives allow the seedlings to survive post-germination, enabling them to be established in the environment and overcome future obstacles during the growth and development of the plant. This offers an explanation as to why these phenylpropanoids are important/at high levels during the early stages of seedling growth, i.e., to act as antimicrobial phytoanticipins. Site-specific hydroxylation, methylation, and glycosylation reactions not only lead to structural diversification within a class of specialized metabolites (and thus increase the ‘chemical space’ thereof) but may also broaden the functional diversification of the molecules. In the later stages, when the plants are more developed and less vulnerable to detrimental environmental conditions, the same metabolic pathways can be (re)activated as part of inducible immunity to provide plant defense responses when needed. Profiling of flavone-based phytoanticipins in juvenile plants may thus be developed as a potential tool for the identification of host resistance.

Overall, the metabolomics investigation substantially extends the knowledge of the metabolite dynamics of early seedling development in sorghum. This affords new insights into the involved metabolic pathways and their regulation through mechanisms not yet documented in the literature that require further investigation.

## Data Availability

Data are contained within the article or supplementary material.

## Supplementary Materials

The following supporting information can be downloaded at: https://www.mdpi.com/article/10.3390/metabo14020112/s1, Figure S1. Growth of *Sorghum bicolor* seedlings; Figure S2. UHPLC-MS BPI chromatograms of methanolic extracts derived from sorghum in ESI negative (A) and ESI positive ionization modes (B); Figure S3. OPLS-DA scores plots of differentially occurring metabolites in extracts of *Sorghum bicolor* seedlings following UHPLC-MS analysis; Figure S4. OPLS-DA S plots of discriminant biomarkers occurring in extracts from *Sorghum bicolor* seedlings following UHPLC-MS analysis; Figure S5. Structures of the flavone, apigenin, and mono- and diglycosylated derivatives thereof. Table S1. Classification and annotation of metabolites from extracts of *Sorghum bicolor* seedlings from different development stages. Table S2. Pathway enrichment analysis based on the presence of metabolites present in hydromethanolic extracts from developing *Sorghum bicolor* seedlings; Table S3. Retention times and MRM/MS data of the precursor ions, product ions, dwell times and collision energies of the standard compounds; Table S4. Standard curve equations and R^2^ values of the standard compounds.
