# Supplementary material for: Data-Driven Characterization of Metabolome Reprogramming during Early Development of Sorghum Seedlings
Source: Metabolites. 2024 Feb 7;14(2):112. doi: 10.3390/metabo14020112 (PMC10891503; doi:10.3390/metabo14020112)
Supplement: Supplementary file 1 [file metabolites-14-00112-s001.zip › metabolites-2824555-supplementary.pdf]

Supplementary Data File

Data-driven characterisation of metabolome reprogramming during early development of sorghum seedlings

Ian A. Dubery *, Lerato P. Nephali, Fidele Tugizimana, Paul A. Steenkamp.

Research Centre for Plant Metabolomics, Department of Biochemistry, University of Johannesburg, P.O. Box 524, Auckland Park 2006, South Africa

---

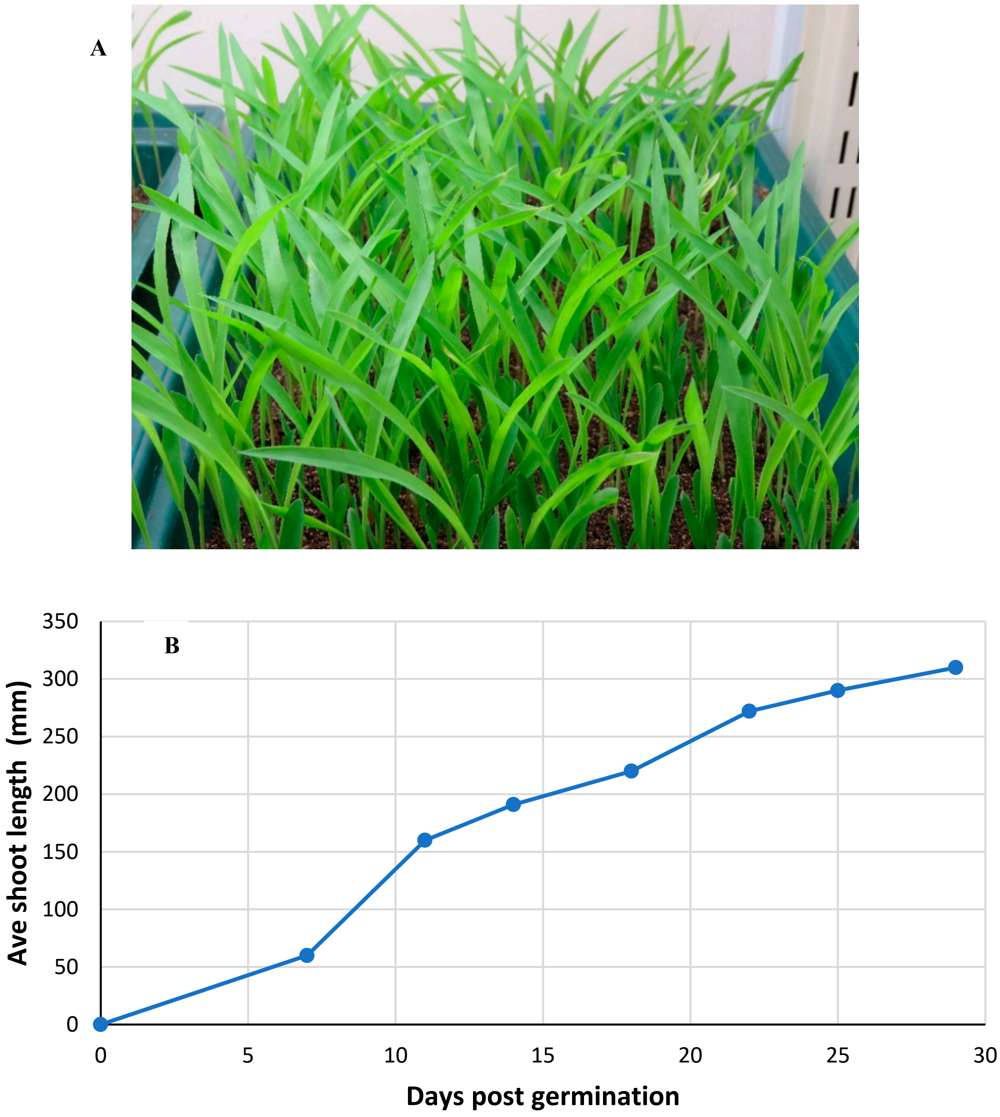

Figure S1. Growth of *Sorghum bicolor* seedlings cv. NS 5511. (**A**) Sorghum seedling growth at day 7 post germination. (**B**) Graph depicting average shoot length at different days.

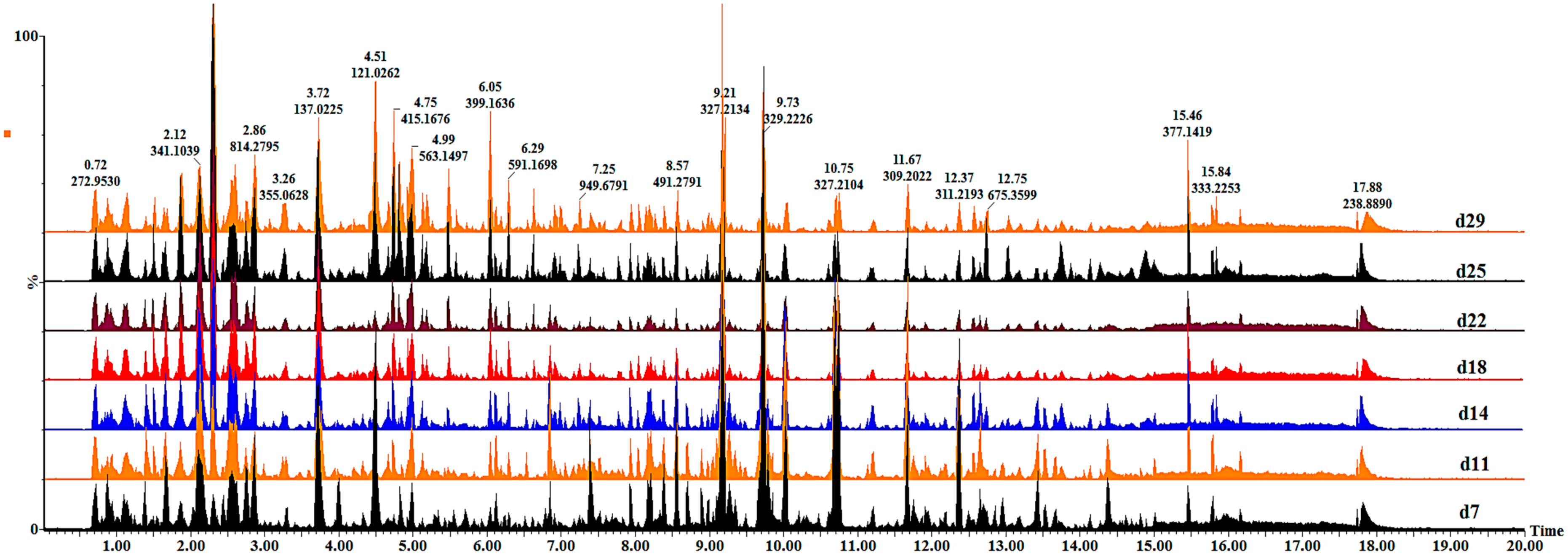

Figure S2A. UHPLC-MS base peak intensity (BPI) chromatograms from methanolic extracts derived from sorghum seedlings in ESI negative ionisation mode. Sample extracts were prepared from plant material harvested at the indicated time intervals. Variation in the displayed chromatograms, linked to changes in the metabolite composition at different developmental stages, can be visually observed from d 7 to d 29.

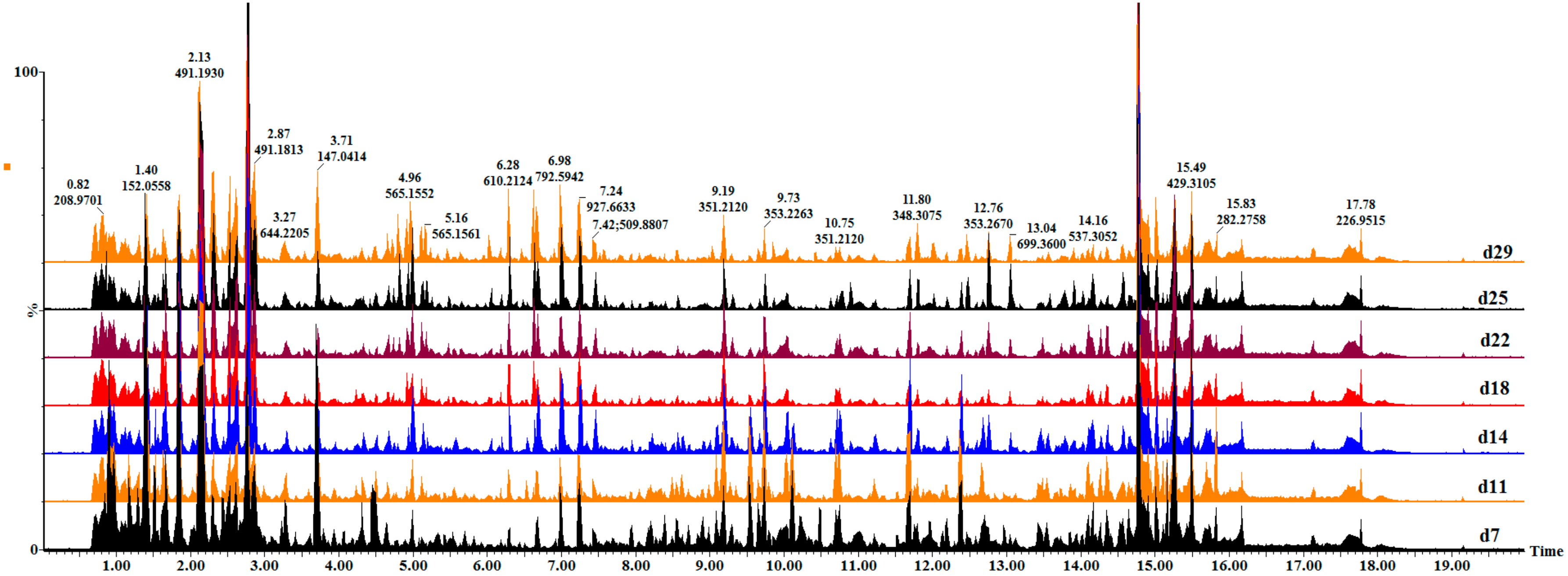

Figure S2B. UHPLC-MS BPI chromatograms of methanolic extracts derived from sorghum seedlings in ESI positive ionisation mode. Sample extracts were prepared from plant material harvested at the indicated time intervals. Variation in the displayed chromatograms, linked to changes in the metabolite composition at different developmental stages, can be visually observed from d 7 to d 29.

Table S1. Classification and annotation of metabolites from leaf extracts of *Sorghum bicolor* seedlings from different development stages (days 7, 14 and 29 post-germination).

Metabolite	*m/z*	Rt (min)	Adduct	Molecular formula	*p*-value **	Fold change ***	Stage (Early, Mid, Late)
**Amino acids and derivatives**							
Phenylalanine	180.092	2.59	$[M-H\_NH_3]^-$	$C_9H_{11}NO_2$	6.71×10^{-6}	0.6	E,M,L
Tyrosine	182.081	1.13	$[M+H]^+$	$C_9H_{11}NO_3$	1.77×10^{-31}	2.1	E,M,L
Dhurrin	334.090	2.60	$[M+H\_Na]^+$	$C_{14}H_{17}NO_7$	2.49×10^{-29}	1.4	E,M,L
Tryptophan	205.097	2.69	$[M+H]^+$	$C_{11}H_{12}N_2O_2$	4.47×10^{-11}	3.6	E,M,L
**Organic acids**							
Citric acid / Isocitric acid	191.018	1.10	$[M-H]^-$	$C_6H_8O_7$	2.60×10^{-6}	0.7	E,M,L
Benzoic acid	121.028	4.46	$[M-H]^-$	$C_7H_6O_2$	1.76×10^{-20}	0.6	E,L
**Flavonoids**							
Apigenin 8-*C*-glucoside (vitexin)	431.099	5.55	$[M-H]^-$	$C_{21}H_{20}O_{10}$	7.57×10^{-5}	1.4	E,M,L
Apigenin 7-*O*-glucoside (apigetrin)	431.098	6.33	$[M-H]^-$	$C_{21}H_{20}O_{10}$	2.38×10^{-3}	1.0	E,M,L
Apigenin 6-*C*-xyloside-8-*C*-glucoside (vicenin-1)	563.142	4.87	$[M-H]^-$	$C_{26}H_{28}O_{14}$	2.01×10^{-4}	2.7	E,M,L
Apigenin 6,8-di-*C*-glucoside (vicenin-2)	593.151	4.45	$M-H]^-$	$C_{27}H_{30}O_{15}$	2.00×10^{-5}	2.7	E,M,L
Apigenin 6-*C*-glucosyl-8-*C*-xyloside (vicenin-3)	563.139	5.09	$[M-H]^-$	$C_{26}H_{28}O_{14}$	1.03×10^{-8}	2.7	E,M,L
Apigenin 7-*O*-neohesperidoside (rhoifolin)	577.156	6.06	$[M-H]^-$	$C_{27}H_{30}O_{14}$	3.58×10^{-8}	3.8	E,M,L
Luteolin 7-*O*-glucoside (luteoloside)	447.091	5.71	$[M-H]^-$	$C_{21}H_{20}O_{11}$	4.44×10^{-13}	1.6	E,M,L
Luteolin 7-*O*-neohesperidoside (lonicerin)	593.179	5.51	$[M-H]^-$	$C_{27}H_{30}O_{15}$	2.20×10^{-15}	1.3	E,M,L
Naringenin 7-*O*-β-D-glucoside (prunin)	433.114	5.91	$[M-H]^-$	$C_{21}H_{22}O_{10}$	1.44×10^{-16}	2.3	M,L
Naringin	625.175	3.33	$[M-H\_FA]^-$	$C_{27}H_{32}O_{14}$	8.31×10^{-9}	30.6	E,M,L
Sophoraflavanone G	423.182	5.46	$[M-H]^-$	$C_{25}H_{28}O_6$	1.21×10^{-10}	30.6	M,L
Quercetin 3-rhamnoside-7-rhamnoside	595.165	4.51	$[M-H]^-$	$C_{27}H_{32}O_{15}$	2.87×10^{-9}	1.4	E,M,L
Quercetin 3-*O*-rhamnoside (quercitrin)	447.092	4.61	$[M-H]^-$	$C_{21}H_{20}O_{11}$	9.02×10^{-17}	1.8	E,M,L
Hesperidin	609.181	4.80	$[M-H]^-$	$C_{28}H_{34}O_{15}$	1.18×10^{-10}	6.5	E,M,L
Unknown flavonoid	581.149	4.33	$[M-H]^-$	$C_{26}H_{30}O_{15}$	6.18×10^{-15}	55.1	E,M,L
Unknown flavonoid	611.158	3.10	$[M-H]^-$	$C_{27}H_{32}O_{16}$	6.18×10^{-15}	55.1	E,M,L
**Hydroxycinnamic acids and derivatives**							
7-Hydroxycoumarin	161.024	1.87	$[M-H]^-$	$C_9H_6O_3$	3.91×10^{-12}	1.3	E,M,L
p-Coumaric acid	163.039	3.65	$[M-H]^-$	$C_9H_8O_3$	$6.40 X 10^{-2}$	0.9	E,M,L
Coumaroyl glucose	327.107	7.22	$[M+H]^+$	$C_{15}H_{18}O_8$	1.00×10^{-4}	2.1	E,M,L
Caffeic acid derivative	475.143	1.92	$[M-H]^-$	$C_{20}H_{28}O_{13}$	1.81×10^{-15}	1.5	E,M,L
2-*O*-Caffeoylglyceric acid	267.048	4.38	$[M-H]^-$	$C_{12}H_{12}O_7$	1.48×10^{-3}	0.5	E,M,L
3-Feruloylquinic acid	367.099	3.75	$[M-H]^-$	$C_{17}H_{20}O_9$	5.98×10^{-9}	1.3	E,M,L
Coniferyl acetate	221.081	7.42	$[M-H]^-$	$C_{12}H_{14}O_4$	4.67×10^{-6}	2.7	E,M,L
Sinapoyl (sinapyl/sinapic) alcohol	209.074	6.72	$[M-H]^-$	$C_{11}H_{14}O_4$	1.11×10^{-7}	0.5	E,M,L
Sinapaldehyde glucoside I	371.130	6.53	$[M+H]^+$	$C_{17}H_{22}O_9$	4.70×10^{-29}	4.3	E,M,L

1,3-*O*-Coumaroyl-feruloylglycerol	413.121	9.03	[M-H]⁻	$C_{22}H_{22}O_8$	9.84×10^{-12}	0.4	E,M,L
Sinapaldehyde glucoside II	415.125	5.68	[M+FA-H]⁻	$C_{17}H_{22}O_9$	6.87×10^{-2}	3.8	E,M,L
**Fatty acids and derivatives**							
Octadecatetraenoic acid (OTA)	275.199	13.44	[M-H]⁻	$C_{18}H_{28}O_2$	1.64×10^{-17}	0.5	E,M,L
15-Hydroxylinoleic acid/15-Hydroxy-9,12-octadecadienoic acid	295.226	14.30	[M-H]⁻	$C_{18}H_{32}O_3$	5.10×10^{-3}	1.2	E,M,L
9,14-Dihydroxy-10,12-octadecadienoic acid (ODA-2OH)(II)	311.219	11.81	[M-H]⁻	$C_{18}H_{32}O_4$	5.87×10^{-17}	0.5	E,M,L
9,12,13-Trihydroxy-10-octadecenoic acid (ODA-3OH)(IV)	329.229	9.60	[M-H]⁻	$C_{18}H_{34}O_5$	1.45×10^{-5}	0.6	E,M,L
Trihydroxyoctadecadienoic acid II (ODA-3OH)(II)	327.213	11.05	[M-H]⁻	$C_{18}H_{32}O_5$	1.54×10^{-5}	0.5	E,M,L
**Phytohormones and derivatives**							
Indole-3-acrylic acid/ N-Ac-indole-3-carboxaldehyde	188.076	2.71	[M+H]⁺	$C_{11}H_9NO_2$	2.27×10^{-10}	3.7	E,M,L
Indole-3-acetyl-leucine	333.120	3.25	[M+H_NaNa]⁺	$C_{16}H_{20}N_2O_3$	9.16×10^{-11}	7.8	E,M,L
Salicylic acid	137.031	3.69	[M-H]⁻	$C_7H_6O_3$	1.50×10^{-1}	0.9	E,M,L
Salicylic acid 2-*O*-beta-D-glucoside	299.074	1.62	[M-H]⁻	$C_{13}H_{16}O_8$	7.21×10^{-17}	1.7	E,M,L
Zeatin riboside	352.183	3.16	[M+H]⁺	$C_{15}H_{21}N_5O_5$	1.35×10^{-28}	1.2	E,M,L
Abscisic acid	265.155	3.35	[M+H]⁺	$C_{15}H_{20}O_4$	$2{,}34 \times 10^{-9}$	1.8	E,M,L
Traumatic acid	297.129	3.90	[M+H_FA-Na]⁺	$C_{12}H_{20}O_4$	1.3×10^{-10}	1.8	E,M,L
Riboflavin	443.118	5.58	[M-FA-NaNa]⁻	$C_{17}H_{20}N_4O_6$	8.10×10^{-3}	0.3	E,M,L

* Annotation was according to level 2 as stipulated by the Metabolomics Standards Initiative [12].
** The *p*-value indicates the probability of a null hypothesis, that there is no difference between two groups that are being analysed, (7 vs. 14 d, 7 vs. 29 d, and 14 vs. 29 d), with *p*-values < 0.05 indicating the null hypothesis can be rejected.
*** Fold change indicates the relative changes in metabolite concentrations between two groups, based on averaged signal intensities. Fold change values differ depending on the stages compared (e.g. L/E) and only the highest values are reported for increases (>1) and lowest values for decreases (<1).

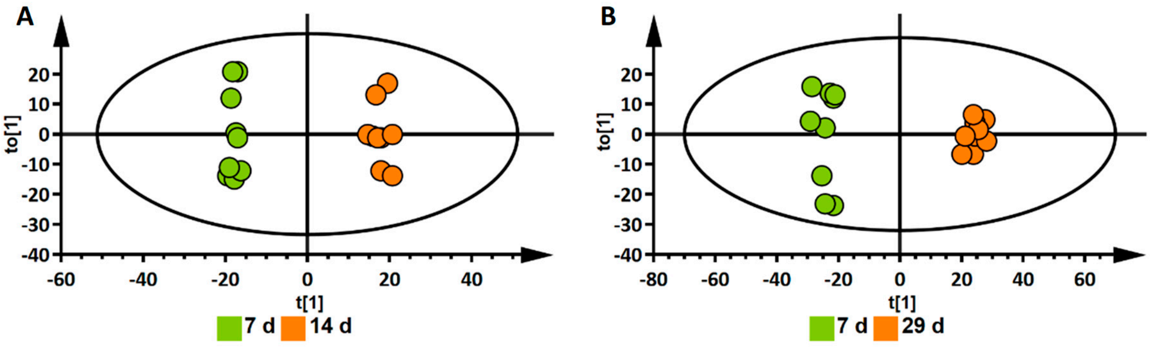

Figure S3. OPLS-DA of differentially occurring metabolites in extracts from *Sorghum bicolor* seedlings following ESI (+/-) UHPLC-MS analysis. OPLS-DA scores plots of the predictive component t[1] and the first orthogonal component t0 [1]. (**A**) 7-d *vs*. 14-d and (**B**) 7-d *vs*. 29-d group samples. The 14-d *vs*. 29-d version of the same plot is presented in the main text.

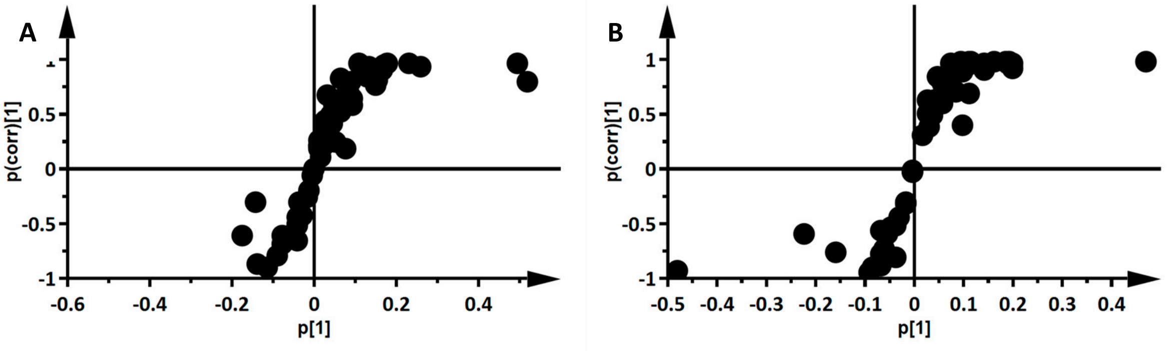

Figure S4. OPLS-DA S plots of discriminant biomarkers occurring in extracts from *Sorghum bicolor* seedlings following ESI (+/-) UHPLC-MS analysis. Discriminant biomarkers are found at each end of the S-plot at **(A)** 7-d *vs*. 14-d and **(B)** 7-d *vs*. 29-d growth stages. The covariance (variable magnitude) and correlation (reliability) of the variables in the model (indicated with black dots), are represented on the axes as p[1] and p(corr)[1] respectively. The *m*/*z* features located at the extreme ends of the plot show a positive association (high magnitude and high reliability) to the respective conditions being compared, while those in the middle can be regarded as shared features.

Table S2. Pathway enrichment analysis based on the presence of metabolites present in hydromethanolic extracts from developing *Sorghum bicolor* seedlings.

Pathway names	Hits	Raw *p**	FDR**	Impact	Matched metabolites
**Arranged by pathway impact > 0.1**					
Biosynthesis of secondary metabolites	1	0.09	1.0	1.00	4-Coumarate
Isoquinoline alkaloid biosynthesis	1	0.10	1.0	0.50	Tyrosine
Phenylalanine metabolism	1	0.18	1.0	0.47	Phenylalanine
Flavone and flavonol biosynthesis	2	0.01	0.42	0.15	Quercitrin, quercetin rhamnoside, glucoside
Tryptophan metabolism	1	0.40	1.0	0.12	Tryptophan
Riboflavin metabolism	1	0.18	1.0	0.12	Riboflavin
Tyrosine metabolism	1	0.25	1.0	0.11	Tyrosine
Glyoxylate and dicarboxylate metabolism	1	0.41	1.0	0.10	Isocitrate
**Arranged by FDR <0.5, raw *p*-value<0.1**					
Phenylpropanoid biosynthesis	4	0.008	0.40	0.06	Phenylalanine, Coumarate, Sinapoyl-OH, Sinapald-Glu
Aromatic amino acids biosynthesis	3	0.006	0.40	0.02	Tyrosine, Tryptophan, Phenylalanine
Flavone and flavonol biosynthesis	2	0.013	0.42	0.15	Quercitrin, quercetin rhamnoside, glucoside
Biosynthesis of secondary metabolites	1	0.087	1.0	1.00	4-Coumarate
Isoquinoline alkaloid biosynthesis	1	0.104	1.0	0.50	Tyrosine
Phenylalanine metabolism	1	0.183	1.0	0.47	Phenylalanine

*Statistical analyses are used to describe these pathways by the *p*-values and false discovery rate (FDR**) of the individual metabolites [19] (Liu *et al.*, 2019). The *p* value was set at < 0.1 and the FDR cut-off was < 0.5.

Table S3. Retention times and multiple reaction monitoring (MRM, MS/MS) data of the precursor ions, product ions, dwell times and collision energies of the standard compounds. Generated quantifier ions resulting from precursor-to-product ion transitions are highlighted in bold.

Compound	Rt (min)	Precursor ions (*m/z*)		Product ions (*m/z*)		Dwell time (msec)	Q1 (V)	CE (V)	Q3 (V)
		[M+H]+	[M–H]-	(+) Mode	(-) Mode				
**Aglycones**									
Naringenin	17.46	273.00	271.00	**163.15*,** 153.05, 147.00	151.05, 119.10, 106.95	100.0	-11.0, -11.0, -11.0	-16.0, -23.0, -19.0	-28.0, -14.0, -14.0
Apigenin	18.23	271.00	269.00	**253.10*,** 239.20, 153.00	151.10, 117.05, 88.95	100.0	-12.0, -10.0, -10.0	-9.0, -9.0, -29.0	-26.0, -16.0, -15.0
Luteolin	16.81	287.00	285.00	**269.20*,** 246.10, 153.05	175.15, 151.15, 132.95	100.0	-23.0, -11.0, -10.0	-8.0, -10.0, -32.0	-19.0, -26.0, -14.0
**Mono-glycosylated derivatives**									
Apigetrin	15.42	433.00	431.00	**400.95*,** 294.95, 271.10	269.15, 268.10, 210.90	100.0	-10.0, -10.0, -10.0	-7.0, -22.0, -22.0	-28.0, -23.0, -28.0
Luteoloside	10.83	449.00	447.00	417.15, **287.15*,** 153.10	379.25, 285.10, 284.15	100.0	-13.0, -11.0, -11.0	-9.0, -21.0, -54.0	-21.0, -19.0, -29.0
Vitexin	9.23	433.00	431.00	**415.10*,** 313.05, 283.00	341.20, 311.15, 283.05	100.0	-10.0, -10.0, -14.0	-20.0, -29.0, -33.0	-20.0, -20.0, -12.0
Iso-vitexin	9.27	433.00	431.00	313.15, 283.05, **200.60***	341.00, 311.15, 283.10	100.0	-10.0, -10.0, -17.0	-25.0, -25.0, -45.0	-21.0, -29.0, -16.0
**Di-glycosylated derivatives**									
Vicenin-2	4.61	595.00	593.00	**475.25*,** 379.20, 324.90	353.25, 383.25	100.0	-20.0, -22.0, -22.0	-17.0, -30.0, -35.0	-16.0, -27.0, -22.0
Vicenin-3	10.88	565.00	563.00	**547.20*,** 457.25, 325.20	473.20, 383.05, 353.00	100.0	-22.0, -20.0, -20.0	-17.0, -19.0, -39.0	-26.0, -16.0, -22.0
**Internal Standard** D-Fluorophenylalanine	1.54	184.00	-	**138.15*,** 118.15, 91.15	-	100.0	-12.0, -11.0, -10.0	-14.0, -22.0, -30.0	-26.0, -11.0, -17.0

*Automated optimisation of MRM parameters was conducted in triplicate for each compound to determine the optimum ionisation polarity of targets. This was followed by optimisation of MRM transitions using LabSolutions software (Shimadzu, Kyoto, Japan). Analyses were performed on a triple quadrupole mass spectrometer equipped with an ESI source operating in both positive and negative ionization modes. LC–MS/MS data were collected and processed by LabSolutions software. The MRM mode was used to quantify the analytes: the assay of investigated compounds was performed following three transitions per compound, the quantifier ions are highlighted in bold and the second and/or the third ones for confirmation.

Table S4. Standard curve equations and R^2 values of the standard compounds used for construction of calibration curves.

Compound	Standard Curve Equation	Regression (R^2)
Apigenin	2E+06x + 106218	0.998
Apigetrin	5E+06x + 782269	0.997
Isovitexin	3E+06x +82308	0.999
Luteolin	5E+06x + 36600	0.990
Luteoloside	4E+06x + 282335	0.998
Naringenin	3E+06x + 178602	0.999
Vicenin-2	133667x + 145.48	0.997
Vicenin-3	811914x - 20143	0.999
Vitexin	1E+06x + 8768.9	0.999
D-Fluorophenylalanine (Internal standard)	1E+06x + 190319	0.990

Each calibration curve for the standards included a working concentration range of 0.05 ppm – 5 ppm within which sample concentrations were obtained. The R^2 (regression) is a measure of the linearity of the standard curve by determining the correlation between the independent (x-axis) and dependent (y-axis) variables. The R^2 values of the calibration curves ranged from 0.990 to 0.999 which is a satisfactory indication of linearity.

Figure S5. Structures of the flavone, apigenin, and mono- and di-glycosylated derivatives, substituted at either O or C. The numbers in the rectangles refer to the unique KEGG identifiers. **Table 1** (main text) lists apigenin-8-*C*-glucoside (vitexin), apigenin-7-*O*-glucoside (apigetrin), apigenin-6-*C*-glucoside-8-*C*-xyloside (vicenin-3), apigenin-6-*C*-glucoside-8-*C*-glucoside (vicenin-2), apigenin 6-*C*-xyloside-8-*C*-glucoside (vicenin-1) and apigenin 7-*O*-neohesperidoside (rhoifolin).

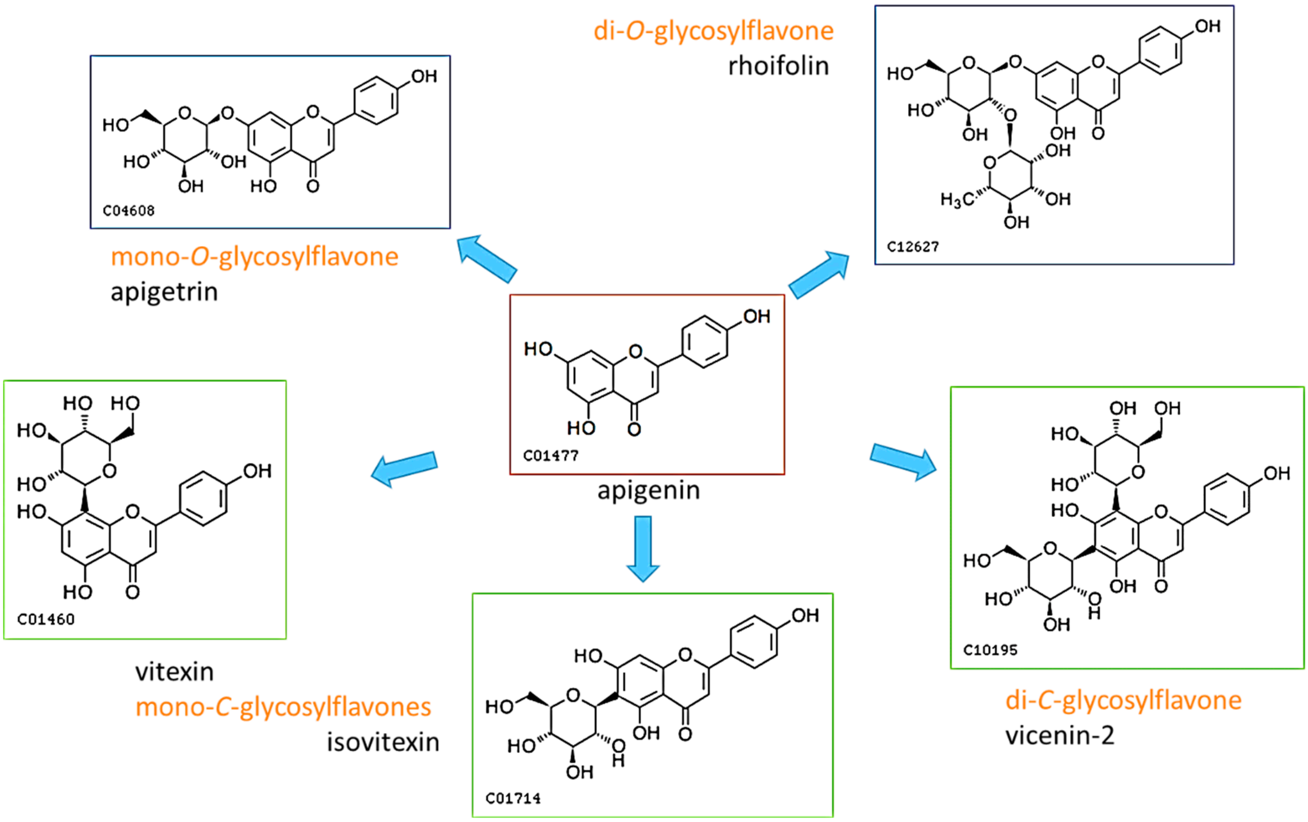

(**Note**: Flavone *C*-glycosides differ from flavone *O*-conjugates in their biosynthetic origin, involving a F2H enzyme (flavanone 2 hydroxylase) that generates isomeric open-ring 2-hydroxyflavanones as substrates for *C*-glycosyl transferases (CGTs) and dehydratases (DHTs) that produce flavone 6- or 8-*C*-glycosides. While the initial steps of the pathway are constitutive, further glycosylation steps may be inducible [50] (Lam, L.P.Y., *et al.* 2023).
